# 148. Antibiotic duration for lower respiratory tract infections with and without procalcitonin monitoring: a propensity score matched cohort across an integrated healthcare network

**DOI:** 10.1093/ofid/ofad500.221

**Published:** 2023-11-27

**Authors:** Daniel J Livorsi, Jamie Heren, Bruce Alexander, Brian Lund

**Affiliations:** University of Iowa Carver College of Medicine, Iowa City, Iowa; Iowa City VA Health Care System, Iowa City, Iowa; Iowa City VA Medical Center, Iowa City, Iowa; Iowa City VA Health Care System, Iowa City, Iowa

## Abstract

**Background:**

Clinical trials have shown that procalcitonin (PCT) testing is efficacious in reducing antibiotic duration, but it is not known whether the test is also effective in routine clinical practice. The goal of this study was to evaluate whether PCT use in patients with lower respiratory tract infections (LRTI) was associated with shorter antibiotic duration.

**Methods:**

We performed a retrospective cohort study across Veterans Affairs acute care hospitals that were ordering PCT with some degree of frequency between January 2018 and December 2021 and included patients with LRTI (acute exacerbations of COPD and pneumonia) that lacked complicating factors (Figure 1). The primary outcome was length of antibiotic therapy. We used 1:1 nearest neighbor propensity score matching to estimate the difference in the primary outcome between PCT-tested and non-tested patients. Patients were fixed matched on hospital site and admission date, and propensity scores were calculated using a logistic regression model that included demographics, ward location, infection type, comorbidities, severity of illness, and antibiotic class.

Figure 1. Flow diagram of patient selection based on inclusion and exclusion criteria

**Figure:**
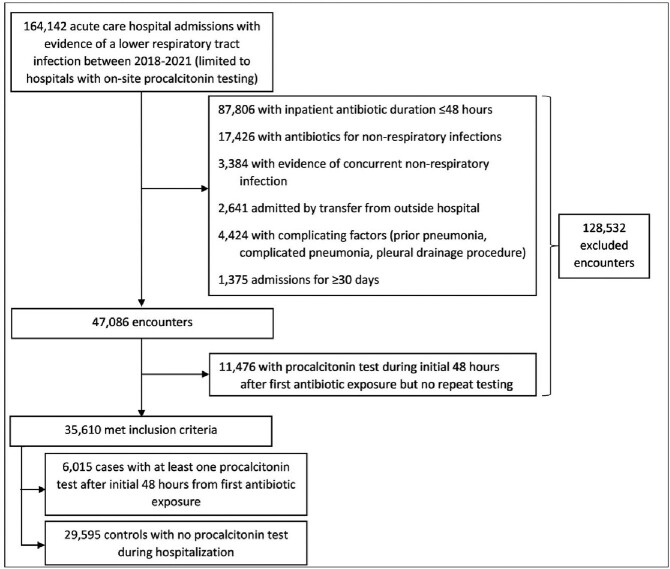

**Results:**

Eighty-one (65%) VA hospitals had on-site PCT testing; all 81 sites had a pharmacist devoting at least some time to antibiotic stewardship activities. Of the 35,610 admissions that met inclusion criteria, 6,015 (17%) had ≥ 1 PCT test at least 48 hours into their antibiotic course (Table 1); in these patients, the median number of PCT tests checked was 2 (interquartile range, 1-3). Using propensity scores, 3,903 patients in the PCT group were matched to 3,903 patients in the no PCT group: the length of therapy was higher in the PCT group compared to the no PCT group with median of 8 days (IQR 6-11) compared to 7 days (IQR 5-10) and mean difference of 0.4 days (p< 0.01, Table 2).

**Figure:**
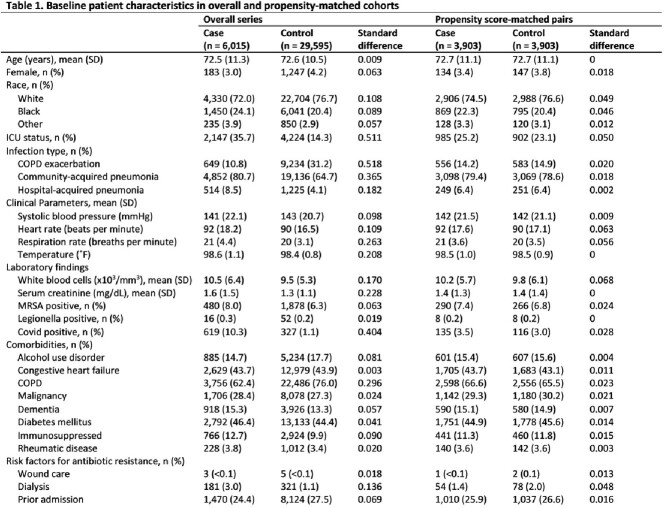

**Figure:**
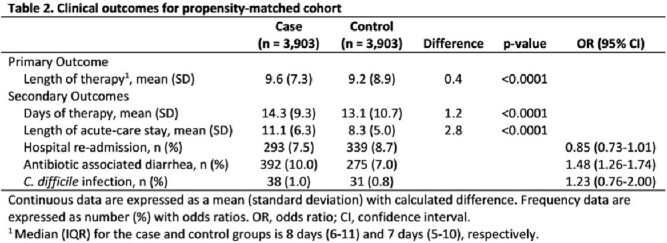

**Conclusion:**

In this retrospective propensity-matched cohort of patients with LRTI across 81 hospitals, we failed to demonstrate shorter antibiotic duration for patients who underwent PCT testing compared to those who did not. While our findings suggest that PCT use may not be effective for reducing antibiotic duration for LRTI under real-life circumstances, it is unclear if the PCT test was optimally implemented at these sites and whether other stewardship activities may have minimized the test's overall impact.

**Disclosures:**

**Daniel J. Livorsi, MD**, Merck: Grant/Research Support

